# Re: Laparoscopic ovarian transposition and ovariopexy for fertility preservation in patients treated with pelvic radiotherapy with or without chemotherapy

**Published:** 2020-05-07

**Authors:** Luz Angela Torres-de la Roche, Rudy Leon De Wilde

**Affiliations:** University Hospital for Gynecology, Pius Hospital Carl von Ossietzky University Oldenburg Georgstrasse 12 - 26121 Oldenburg, Germany.

Dear Editor,

We would like to thank Turkgeldi et al. for their work and publication on “Laparoscopic ovarian transposition and ovariopexy for fertility preservation in patients treated with pelvic radiotherapy with or without chemotherapy” (Turkgeldi et al., 2019).

Radiotherapy to the lower abdomen has been shown to have a significant negative effect on fertility and the hormonal function in women of childbearing age. Radiotherapy is used frequently in the treatment of many malignant conditions in premenopausal women and thereby has eventually a castrating effect.

In the beginning of the nineties, we adapted the open ovariopexy (Grabenbauer et al., 1991) to a minimal access laparoscopic technique dissecting the infundibulopelvic ligament free, high-up cranially to obtain a non-kinked vascular pedicle, which was afterwards fixed outside the true pelvis. The results of our comparative study in 18 patients showed a clear hormonal benefit for these young women, with further reproductive possibilities (De Wilde and Hesseling., 1995).

In order to preserve as much ovarian function as possible, besides the possible pharmacological protective measurements (Von Wolff et al., 2010), the laparoscopic tubo-ovarian transposition with ovariopexy has been shown to be a feasible and effective minimal-access surgical protective step, and could be offered to all above described patients.

Luz Angela Torres-de la Roche, Rudy Leon De Wilde, University Hospital for Gynecology, Pius Hospital Carl von Ossietzky University Oldenburg Georgstrasse 12 - 26121 Oldenburg, Germany. E-mail: luz.angela.torres-de.la.roche@uni-oldenburg.de
